# Near infrared photoimmunotherapy for cancers: A translational perspective

**DOI:** 10.1016/j.ebiom.2021.103501

**Published:** 2021-07-28

**Authors:** Yasuhiro Maruoka, Hiroaki Wakiyama, Peter L. Choyke, Hisataka Kobayashi

**Affiliations:** a Molecular Imaging Branch, Center for Cancer Research, National Cancer Institute, National Institutes of Health, Bethesda, MD 20892, USA; b Departments of Clinical Radiology, Graduate School of Medical Sciences, Kyushu University, Fukuoka 812-8582, Japan

**Keywords:** Near-infrared photoimmunotherapy, Head and neck cancer, Antibodies, Epidermal growth factor receptor, Immune checkpoint inhibitor, Regulatory T cell

## Abstract

Near-infrared photoimmunotherapy (NIR-PIT) is a newly-developed, highly-selective cancer treatment, which utilizes a monoclonal antibody conjugated to a photoabsorbing dye, IRDye700DX (IR700). The antibody conjugate is injected into the patient and accumulates in the tumour. Within 24 h of injection the tumour is exposed to NIR light which activates the conjugate and causes rapid, selective cancer cell death. A global phase III clinical trial of NIR-PIT in recurrent head and neck squamous cell cancer (HNSCC) patients is currently underway. Conditional clinical approval for NIR-PIT in recurrent HNSCC has been granted in Japan as of September 2020. Not only does NIR-PIT induce highly selective and immediate cancer cell killing, but it also stimulates highly active anti-tumour immunity. While monotherapy with NIR-PIT has proven effective it is likely that combinations with immune-checkpoint inhibitors or additional NIR-PIT targeting immune suppressive cells in the tumour microenvironment will further improve results. In this review, we discuss the translational aspects of NIR-PIT especially in HNSCC, and potential future applications.

## 1 Introduction

Near infrared photoimmunotherapy (NIR-PIT) is a newly-developed, highly-selective cancer treatment that induces necrotic/immunogenic cell death. NIR-PIT is a two-part therapy which utilizes (1) a monoclonal antibody (mAb) conjugated to a photoabsorbing dye, IRDye700DX (IR700), which is then (2) activated by NIR light [1,2]. After intravenous injection, each antibody-photoabsorber conjugate (APC) binds to its cognate receptor overexpressed on the surface of cancer cells. Then, NIR light at 690 nm is delivered by laser to excite IR700 on the APC, leading to rapid, highly selective, lethal damage to the cell membrane. NIR-PIT can be targeted with many different antibodies [3–8]. The nature of the cell death associated with NIR-PIT is unique. Cells rapidly swell, bleb and rupture, emptying intracellular contents into the extracellular compartment thus stimulating a vigorous host immune response. A first-in-human phase I/II clinical trial of NIR-PIT using cetuximab-IR700 (RM-1929) targeting epidermal growth factor receptor (EGFR) in patients with inoperable head and neck squamous cell cancer (HNSCC) was successfully completed in late 2017 (https://clinicaltrials. gov/ct2/show/NCT02422979). The phase I/II trials showed NIR-PIT was at least equal if not superior to existing second- and third-line therapies for recurrent HNSCC with fewer adverse events, which suggests that NIR-PIT could be an effective new treatment for HNSCC. A global phase III human clinical trial was initiated in 2018 and is currently underway (https://clinicaltrials.gov/ct2/show/NCT03769506). Recently, NIR-PIT using cetuximab-IR700 was conditionally approved for recurrent HNSCC and was registered for clinical use by the Pharmaceuticals and Medical Devices Agency in Japan. Here, we focus on specific advantages and applications of NIR-PIT.

## 2 Mechanism of action of NIR-PIT

### 2.1 Physicochemical reaction after NIR light exposure

**Fig 1:**
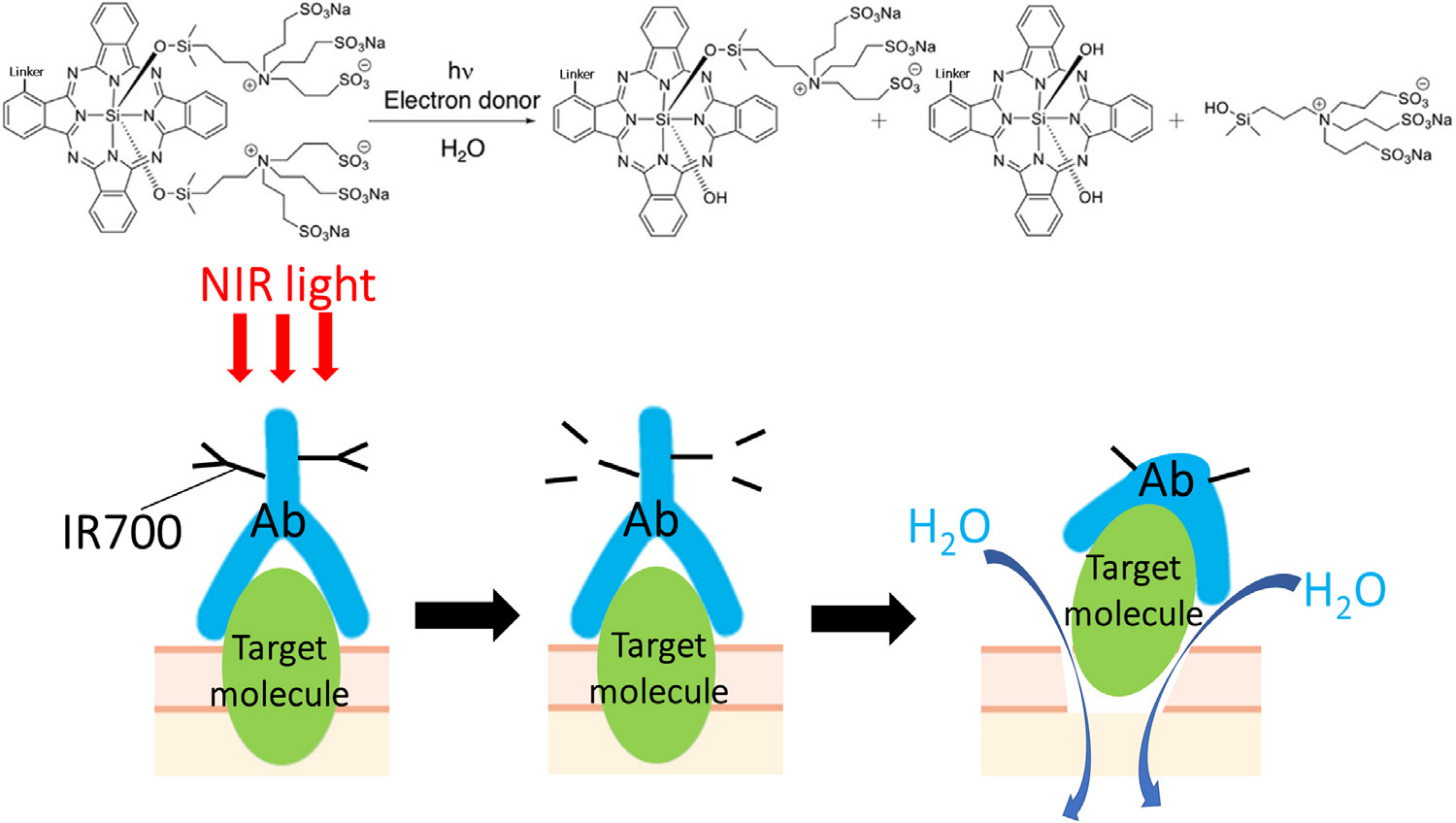
Schematic illustration of the chemical basis of NIR-PIT (upper) and physicochemical changes in APC after NIR light exposure (lower).

The conjugation chemistry to create the APC is straightforward. Briefly, the mAb is incubated with IR700 NHS ester and 0.1 mol/L Na_2_HPO_4_ (pH 8.5) at room temperature for 1 h. The mixture is purified using a gel filtration column. Administration of the APC via intravenous injection leads to binding of the conjugate to the tumour, and is followed by NIR light exposure which induces a photochemical ligand reaction that releases the hydrophilic side chains of IR700 causing the remaining molecule to rapidly become extremely hydrophobic (Fig. 1) [2,9]. IR700 is ordinarily a water-soluble photoreactive dye with no phototoxic or biotoxic properties of its own. Unbound IR700 that dissociates from the APC is readily excreted in the urine without toxic effects [9]. However, APC is a potent killer of cancer cells when activated by NIR light. The APC fluoresces when it is intact. However, as high intensity NIR light is applied, fluorescence begins to decline from the photochemical ligand release within the IR700 molecule leading to photobleaching. Importantly, the photochemical ligand release reaction is accompanied by physical changes in aggregation and solubility within the APC-antigen complex itself. These dramatic photochemical changes damage the APC-bound cell membrane integrity. Damage to the cell membrane impairs cellular membrane function and allows water to rapidly flow into the cell leading to swelling and ultimately, cell rupture and death.

### 2.2 Highly selective cell killing

Selectivity of NIR-PIT cell killing is achieved by targeting overexpressed antigens on the surface of cancer cells, however, little or no damage occurs in surrounding normal cells that do not overexpress the antigen. Thus, NIR-PIT induces selective killing of cancer cells with minimal off-target effects. Additionally, the laser light that is applied is nonthermal and does not use ionizing radiation, thus there is no damage to cellular DNA. As a result, non-specific damage due to the applied light is minimal [1]. Most patients treated with NIR-PIT have significant tumour shrinkage with very few adverse effects. Moreover, unlike most cancer therapies, NIR-PIT can be repeatedly administered if there is residual or recurrent disease. Repeated dosing of APCs and NIR light has been previously reported to improve efficacy of NIR-PIT by increasing the frequency of complete responses in targeted tumours [10].

### 2.3 Anti-tumour immunity enhancement

**Fig 2:**
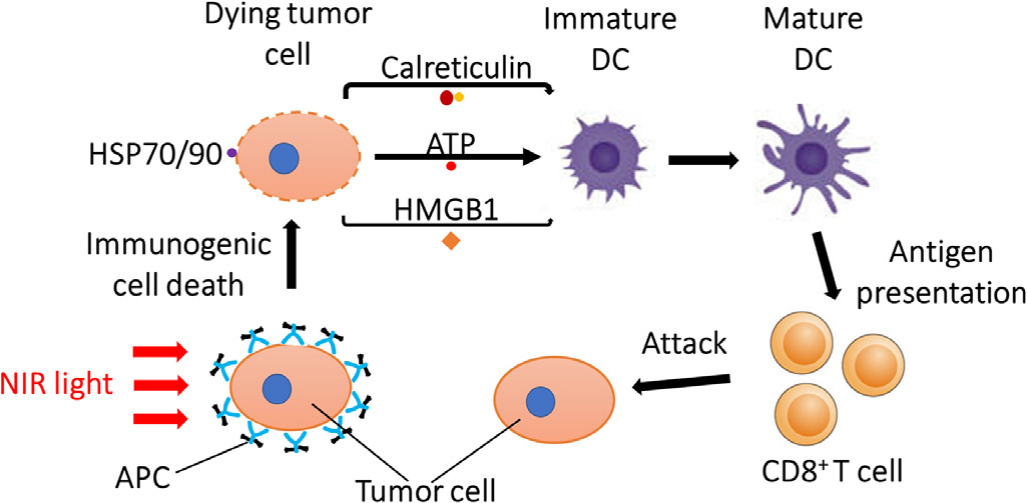
Biological mechanism of ICD induced by NIR-PIT that leads to enhanced antitumor host immunity against treated cancer cells.

Targeted cell death induced by NIR-PIT is immunogenic because there is rapid (within a few minutes of light application) release of antigenic cellular contents including cancer-specific antigens into the tumour microenvironment (TME) [2,9]. NIR-PIT-induced cell rupture can upregulate the expression of heat shock proteins (HSPs) such as HSP70 and HSP90, which can bind tumour antigens and interact with Toll like receptors (TLRs), activating antigen presenting cells. In addition, after NIR-PIT, cells release calreticulin, adenosine triphosphate (ATP), and high-mobility group box 1 (HMGB1) which promote maturation of immature dendritic cells (DCs) in the TME [11]. Mature DCs prime and educate cancer-specific naive T cells, leading to cancer cell killing mediated by proliferation of cytotoxic T cells [11,12]. This process is known as immunogenic cell death (ICD) (Fig. 2). Most cancer therapies induce mostly apoptotic cell death which is an orderly multistep programmed process that takes hours to days culminating in cell death that is minimally immunogenic. Moreover, chemotherapy and radiation also take with them important immune cells in the TME. In the case of NIR-PIT the rapid release of multiple neoantigens leads to newly primed CD8^+^ T cells that can recognize and respond to multiple cancer antigens and then proliferate to become systemic [12]. Therefore, anti-tumour host immunity is enhanced after NIR-PIT primarily by re-education and subsequent expansion of CD8^+^ T cells in the TME, after neoantigens are released . The effect is not only seen in the NIR-PIT treated tumours but also in untreated tumours of a similar type located elsewhere in the body.

### 2.4 Super-enhanced permeability and retention effects

**Fig 3:**
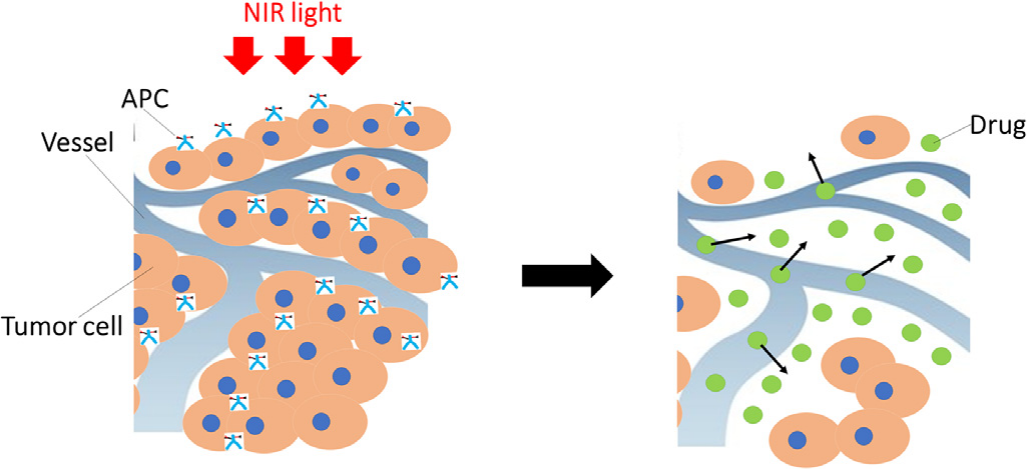
Schematic illustration for mechanism of SUPR effects induced by NIR-PIT.

NIR-PIT has another unique feature related to its effect on tumour vascularity. Some degree of enhanced permeability and retention (EPR) is usually observed in most tumours due to vascular leakiness. However, after NIR-PIT, rapid and marked increases in permeability are observed in the vessels supplying the tumour. Immediate necrosis in the perivascular cancer cells after NIR-PIT produces a potential space between the vessels and the remaining tumour, leading to vessel enlargement, increase in blood volume, and decrease in blood velocity and vascular resistance. The net result is increased permeability to an extent far greater than is seen with the EPR effect [13–15]. For instance, the ability to deliver nano-sized or macromolecular drugs into tumour tissues treated with NIR-PIT increased up to 24-fold compared with non-treated tumours. EPR effects are generally < 2-fold in magnitude. This phenomenon has been called the super-enhanced permeability and retention (SUPR) effect to reflect this large increase in permeability (Fig. 3) [13,14]. The SUPR effect can be exploited to deliver additional therapeutic agents into the treated tissue. Preclinically, CD44-targeted NIR-PIT combined with systemic administration of anti-programmed cell death protein 1 (anti-PD-1) mAb improved treatment outcome compared with CD44-targeted NIR-PIT alone or anti-PD-1 mAb administration alone. The SUPR effect is likely responsible for these synergistic effects because the antibody can be distributed more evenly throughout the tumour due to the SUPR effect [12,16].

## 3 Successful preclinical applications of NIR-PIT

Extensive preclinical studies in tumour-bearing mouse models have been performed [1,2]. Typically cancer therapies are first tested in xenografts in the athymic mouse model, a murine strain with the spontaneous deletion of the Foxn1 gene which prevents T cell production [1]. The absence of T cells reduces the chance that the implanted tumour will be rejected. However, results of NIR-PIT in cancers implanted in athymic nude mice are misleading. Usually, such models underestimate the effect due to the absence of a robust immune response. Syngeneic mouse models, on the other hand, have an intact immune system and are therefore used to investigate the anti-tumour immune response in preclinical studies of NIR-PIT [2]. As long as tumour antigens are overexpressed on cell membranes and antibodies (typically murine) exist for these antigens, NIR-PIT can be applied to virtually any cancer or TME cell. NIR-PIT has been successfully applied for EGFR expressing tumors (e.g., skin cancer [10], lung cancer [17], and breast cancer [8]), human epidermal growth factor receptor 2 (HER2) (e.g., gastric cancer [18] and breast cancer [19]), prostate-specific membrane antigen (PSMA) (e.g., prostate cancer) [20], carcinoembryonic antigen (CEA) (e.g., colon cancer) [21], [22], [23], mesothelin (MSLN) (e.g., mesothelioma, pancreatic, and ovarian cancers) [6], Glypican 3 (GPC3) (e.g., hepatocellular carcinoma) [24], CD20 (e.g., malignant lymphoma) [5], tumour‐associated calcium signal transducer 2 (TROP2) (e.g., cholangiocarcinoma) [25], delta-like protein 3 (DLL3) (e.g., small cell lung cancer) [22], podoplanin (PDPN) (e.g., mesothelioma) [23] and G-protein coupled receptor 87 (GPR87) (e.g., lung cancer and mesothelioma) [24], as shown in the Table 1. NIR-PIT can also be used for programmed death-ligand 1 (PD-L1) expressing cancer cells [26], for CD25 for regulatory T cells (Tregs) [7,27,28], and CD44 [4,12,16,29,30] and CD133 for cancer stem cells [31].

**Table 1:** Target molecules for near-infrared photoimmunotherapy.

Target	Disease	Type of tumor model used	Key findings
Tumor antigen			
EGFR	Head and neck cancer, skin cancer, lung cancer, breast cancer, uterine cervical cancer [8,10,17,49]	Allograft (subctaneous), xenograft (subctaneous, orthotopic)	Tumour volume ↓, Survival ↑, Tumour immunity ↑
HER2	Gastric cancer, breast cancer [18,19]	Xenograft (subctaneous, disseminated peritoneal models)	Tumour volume ↓, Survival ↑
PSMA	Prostate cancer [20]	Xenograft (subctaneous)	
CEA	Colon cancer, biliary tract cancer, pancreatic cancer [21], [22], [23]	Xenograft (subctaneous, orthotopic)	
MSLN	Mesothelioma, pancreatic cancer, ovarian cancer [6]	Xenograft (subctaneous)	
GPC3	Hepatocellular carcinoma [24]	Xenograft (subctaneous)	
CD20	B-cell malignant lymphoma [5]	Xenograft (subctaneous)	
TROP2	Colon cancer, gastric cancer, pancreatic cancer [25]	Xenograft (subctaneous)	
DLL3	Small cell lung cancer [22]	Xenograft (subctaneous)	
PDPN	Mesothelioma [23]	Xenograft (subctaneous, orthotopic)	
GPR87	Lung cancer and mesothelioma [24]	Xenograft (subctaneous, disseminated pheural models)	
PD-L1	Lung cancer [26]	Xenograft (subctaneous)	
Cancer stem cell			
CD44	Colon cancer, lung cancer, head and neck cancer [4, 12, 16, 29, 30]	Allograft (subctaneous, orthotopic), xenograft (subctaneous)	Tumour volume ↓, Survival ↑, Tumour immunity ↑
CD133	Glioblastoma [31]	Xenograft (subctaneous, orthotopic)	Tumour volume ↓, Survival ↑
Immune cell			
CTLA4	Colon cancer, lung cancer, head and neck cancer [62]	Allograft (subctaneous)	Tumour volume ↓, Survival ↑, Tregs ↓, Tumour immunity ↑
CD25	Colon cancer, lung cancer, head and neck cancer [7,27]	Allograft (subctaneous)	

EGFR, epidernal growth factor receptor; HER2, human epidermal growth factor receptor-2; PSMA, prostate-specific membrane antigen; CEA, carcinoembryonic antigen; MSLN, mesothelin; GPC3, glypican-3; TROP2, tumor-associated calcium signal transducer 2; DLL3, delta-like protein 3; PDPN, podoplanin; GPR87, G protein-coupled receptor 87; PD-L1, programmed death-ligand 1; CTLA4, cytotoxic T-lymphocyte-associated protein 4; Tregs, regulatory T cells. The numbers in the brackets show reference number

A limitation of NIR-PIT is that it must be activated by NIR light. NIR light can penetrate tissue to a depth of approximately 2 cm from the surface. However, more deeply located tumours require interstitial NIR light exposure in which fibre-optical diffusers are inserted through catheter needles into the substance of the tumour [32,33]. The combination of external and interstitial delivery of NIR light is common in large tumours, and this deployment of light results in a more homogeneous light distribution. Additionally, light can be delivered via endoscopic optical fibre diffusers to approach tumours in the gastrointestinal tract or urinary tract [18]. NIR light delivery during endoscopic procedures enables the operator to treat tumours under direct vision. Interstitial or endoscopic NIR-PIT can be readily adapted to clinical practice and are used in human clinical trials of NIR-PIT for HNSCC patients. If NIR-PIT is combined with surgical resection, NIR light can be directed to the surgical margins periodically throughout the procedure to reduce the chance of recurrence [34].

Circulating tumour cells (CTCs) are thought to be important in promoting metastases. CTCs circulate in the blood vessels until they successfully engraft at sites whereupon they recruit other stromal cells important for the development of metastases. CTCs can express cell surface markers that may be targeted by NIR-PIT using specific APCs while they are circulating and not yet visibly metastatic. Continuous NIR light irradiation of superficial blood vessels, such as those in the wrists and neck, might reduce absolute numbers of CTCs and improve survival. In the near future, it is expected that CTC-targeted NIR-PIT may be a method of prolonging disease-free survival.

NIR-PIT also has potential to improve tissue engineering. During tissue engineering, stem cells are placed on specific scaffolds which allow them to grow into replacement organs or organoids that can subsequently be functional *in vivo*. During the growth of these new tissues, teratomas may develop rendering the graft useless. NIR-PIT could be used to eliminate teratomas without damage to the remaining graft and thus save the graft from being discarded [35,36].

## 4 Translational application of NIR-PIT

### 4.1 HNSCC

Cancer is a leading cause of death worldwide [37], and HNSCC is the sixth most common cancer [38]. Up to 90 % of head and neck tumours are squamous cell carcinomas [39]. Three major types of cancer therapy, surgery, radiation, and chemotherapy, have been traditionally used to treat HNSCC. These treatments kill or remove cancer but also cause substantial damage to normal critical surrounding tissues, leading to marked reductions in quality of life. Despite a variety of innovative treatment strategies for HNSCC, including molecularly targeted therapy and immunotherapies, the overall 5-year survival rate remains approximately 60 % [40,41]. Approximately 30–50% of HNSCC patients with locally advanced tumours have local recurrence or disease progression after initial therapy [42,43]. Local tumour growth can cause severe symptoms, including pain, difficulty of breathing, speaking, and swallowing. New types of cancer treatments that improve the clinical outcomes of HNSCC patients without unacceptable side effects are urgently needed.

### 4.2 EGFR-targeted NIR-PIT

EGFR is one of the most ubiquitous cancer cell surface receptors and is overexpressed on skin, lung, colon, head and neck, breast, and oesophageal cancers [44]. It is a member of the erb-b2 receptor tyrosine kinase (ERBB) family of receptor tyrosine kinases that includes ERBB2-4. Activation of EGFR [46] promotes proliferation, invasion, angiogenesis, and metastasis [47,48] EGFR is overexpressed in up to 90% of primary HNSCC [45] and is the target of several types of drugs which block ligand binding, inhibit EGFR tyrosine kinases or induce antibody dependent cellular cytotoxicity. Three human EGFR-targeting mAbs, cetuximab, panitumumab, and necitumumab, have been approved by the FDA. Of these, cetuximab, a chimeric mAb, and panitumumab, a human mAb, have been widely used in EGFR-expressing cancers. In xenograft models, human EGFR-targeted NIR-PIT greatly inhibits tumour growth and prolongs survival compared with EGFR mAb monotherapy in various types of cancer cells [8,10,17]. Moreover, NIR-PIT with cetuximab-IR700 or panitumumab-IR700 in a murine oropharyngeal cancer cell line decreased tumour progression and prolonged survival significantly in part due to enhanced host immunity [49]. Thus, EGFR is an important target of HNSCC for NIR-PIT. A global Phase III multi-centre clinical trial with ASP-1929 (anti-EGFR antibody cetuximab conjugated to IR700, analogous to RM-1929) in patients with recurrent HNSCC who have failed at least two lines of therapy is ongoing (https://www.clinicaltrials.gov/ct2/show/NCT03769506).

### 4.3 NIR-PIT combined with immune checkpoint inhibitor

**Fig 4:**
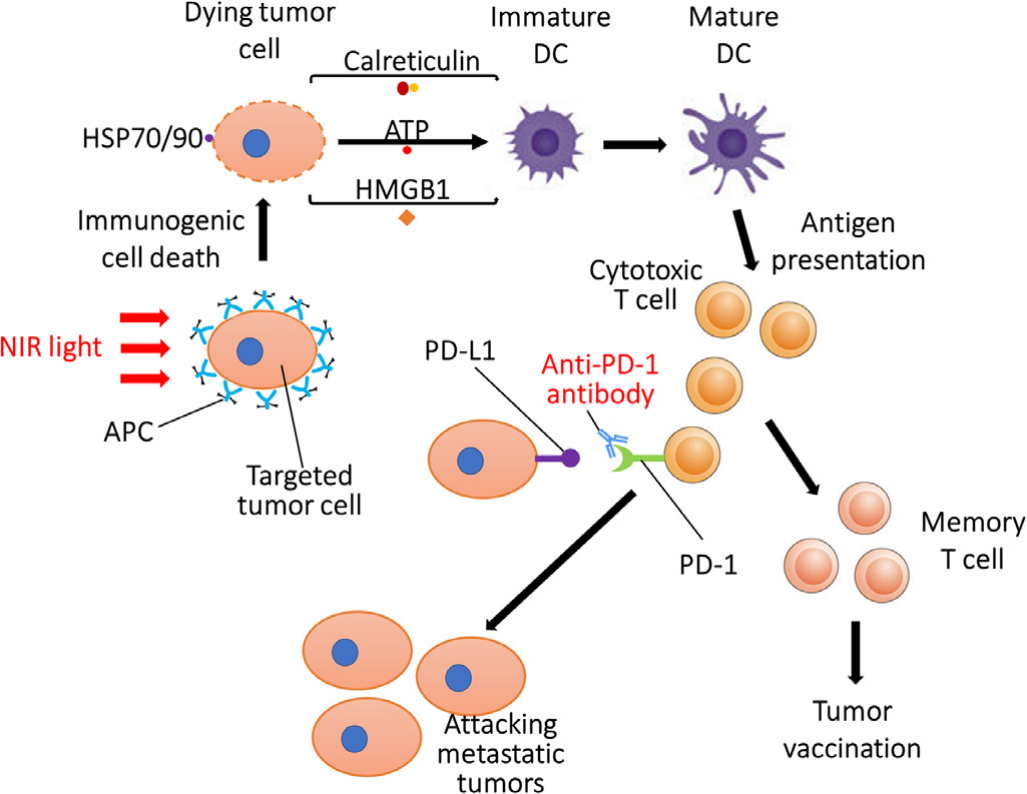
Proposed mechanism of combination therapy with NIR-PIT and systemic administration of anti-PD-1 monoclonal antibody.

CD44 is a well-known marker of cancer stem cells and is implicated in intercellular adhesion, cell migration, cell spatial orientation, and promotion of matrix-derived survival signal [50]. Cell transformation, uncontrolled cell growth, resistance to apoptosis, and active cell migration are mediated by CD44 along with other factors [51]. High expression of CD44 is associated with tumour aggressiveness and poor outcome and CD44 inhibition can impair tumour growth [52]. CD44-positive cancer stem-like cells in HNSCC have the capacity for tumour initiation and long-term self-renewal. These cells can also evade host immune responses by inducing expression of PD-L1 [53]. Thus, CD44 antigen is an important target for antibody-based therapies. In preclinical studies in mice, CD44-targeted NIR-PIT, combined with an immune checkpoint inhibitor, achieved complete tumour killing including metastatic lesions that were not exposed to NIR light. The mice that have experienced complete response rejected any attempts to re-inoculate the tumour a month after NIR-PIT, suggesting that these mice had gained immunity against the initial tumour (Fig. 4) [12,16]. NIR-PIT can also convert a minimally immunogenic tumour, unresponsive to immune checkpoint inhibitor, into a highly immunogenic tumour responsive to immune checkpoint inhibitors [16]. When NIR-PIT is used in combination with an immune checkpoint inhibitor, systemic administration of immune checkpoint inhibitor was required only for approximately one week to achieve complete remission of targeted and distant lesions after NIR-PIT. The short-term use of immune checkpoint inhibitor may help minimize adverse effects caused by the typical long-duration systemic administration of immune checkpoint inhibitors. Clinical trials with ASP-1929 photoimmunotherapy in combination with anti-PD-1 therapy in recurrent/metastatic HNSCC or advanced/metastatic cutaneous SCC (https://www.clinicaltrials.gov/ct2/show/NCT04305795) and in recurrent/metastatic gastric or oesophageal cancer (https://clinicaltrials.gov/show/JapicCTI-194969) are currently underway.

### 4.4 Treg depletion by CD25-targeted NIR-PIT or CTLA4-targetd NIR-PIT

**Fig 5:**
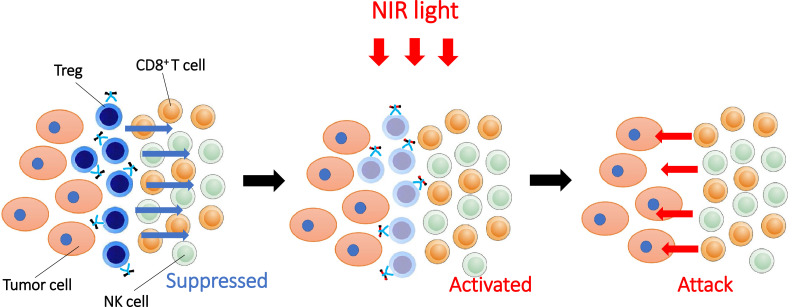
Enhancement of systemic antitumor host immunity induced through selective depletion of Treg by NIR-PIT.

Immunosuppressive cells within the TME are promising targets for NIR-PIT. Tregs are crucial for immunological self-tolerance and help maintain immune homeostasis and prevent autoimmune disease [54]. Tregs are widely regarded as one of the primary mediators of immune tolerance [55]. Tregs induce immunosuppression using a variety of mechanisms including inhibiting interleukin-10 (IL-10) and transforming growth factor beta (TGFβ), suppression of natural killer (NK) cells and effector T cells through secretion of cytotoxic substances, suppression by metabolic disruption of effector T cells due to consumption of interleukin-2 (IL-2), and interfering with DC function [56]. Tregs thus, possess highly immunosuppressive functions and high ratios of Tregs to CD8^+^ T cells among tumour-infiltrating lymphocytes (TILs) are associated with a poor prognosis. The degree of Treg infiltration in the TME correlated with prognosis in HNSCC [57,58]. Although a variety of methods for Treg depletion have been previously attempted [59], systemic administration of anti-CD25-IgG reportedly depletes peripheral Tregs, but may induce autoimmune adverse events such as cytokine storm. NIR-PIT, however, can selectively deplete Tregs only within the TME without eliminating local effector T cells or Tregs located in other organs. CD25 is a component of the IL-2 receptor complex (IL-2Rs) and is mainly expressed on Tregs and to a lesser extent on activated effector cells, such as CD8^+^ T cells and NK cells [60,61]. Thus, CD25-targeted NIR-PIT selectively depleted Tregs from the TME, resulting in activation of effector cells and upregulation of anti-tumour immunity (Fig. 5). Local CD25-targeted NIR-PIT depleted Tregs only at the tumour site where NIR light was applied and therefore, could be a safer method than systemic Treg depletion. In CD25-targeted NIR-PIT, there is theoretical concern about depleting activated effector T cells either due to antibody-dependent cellular cytotoxicity (ADCC)/complement-dependent cytotoxicity (CDC) and/or reduction of activated effector T cell function by blocking IL-2 binding. To overcome this limitation, a truncated antibody [anti-CD25-F(ab′)_2_] generated by removal of the Fc region of an anti-CD25-IgG was used for local Treg depletion. The smaller size of the F(ab′)_2_ also resulted in more rapid clearance and less blocking of IL-2/IL-2R binding on effector cells, thus, did not interfere with the desired immune response [7,27]. Local CD25-targeted NIR-PIT induced rapid activation of tumour-infiltrating CD8^+^ T cells, NK cells and antigen-presenting cells and greatly enhanced host immunity against both targeted and non-targeted lesions [7,27]. The effect of Treg depletion by NIR-PIT continued for 3 to 4 days and thereafter there was a gradual repopulation of Tregs, reaching pre-treatment numbers of Tregs about 6 days after therapy [7]. However, NIR-PIT can be repeatedly performed if tumours recur, and repeated Treg cell depletion with CD25-targeted NIR-PIT can induce prolonged tumour control and survival. In addition to CD25-targeted NIR-PIT, T lymphocyte associated protein 4 (CTLA4)-targeted NIR-PIT can also help eliminate Tregs because CTLA4 is expressed on Tregs [62]. CTLA4-targeted NIR-PIT reduced tumour progression and prolonged survival in mouse tumour models. After CTLA4-targeted NIR-PIT, activation and infiltration of CD8^+^ T cells was observed in the treated tumour beds [62].

NIR-PIT is capable of simultaneously targeting two or more kinds of cells with a single shot of NIR light exposure simply by co-injecting two or more different APCs followed by exposure to NIR light [63,64]. In other words, NIR-PIT can simultaneously eliminate both cancer cells and Tregs in a single treatment. CD25-targeted NIR-PIT with CD44-targeted NIR-PIT resulted in a substantial complete response rate in CD44 overexpressing tumour models [28]. Moreover, simultaneous EGFR-targeted NIR-PIT with CD25-targeted NIR-PIT inhibited tumour growth and prolonged survival compared to monotherapy in allografted mouse models [47]. Therefore, the combination of NIR-PIT targeting cancer cells and Tregs have potential to become clinical treatments for HNSCC.

### 4.5 Additional preclinical tumour models for NIR-PIT

Due to the limited number of available murine monoclonal antibodies against syngeneic tumours, CD44, a cancer stem cell marker, was initially used as a tumour target. However, because CD44 is also expressed on effector T cells there are disadvantages to its use. Therefore, as an alternative, a human EGFR-expressing mouse oral squamous cell cancer model, mEERL-hEGFR, human EGFR-expressing murine oropharyngeal cancer cell, was established and was used for combination therapy of human EGFR-targeted NIR-PIT with CD25-targetd NIR-PIT [47], which is exactly the regimen that is planned for a clinical trial.

Additionally, in order to further simulate the clinical setting, orthotopic mouse models would be superior to xenografted tumour models so that the TME is more realistically recapitulated [63]. *In vivo* therapeutic efficacy and immune response to NIR-PIT in orthotopic mouse models of a syngeneic head and neck tumour have been demonstrated [65].

### 4.6 NIR-PIT targeting artificially induced antigens

Gene transfer technology can be used to make cancer cells express artificial targets suitable for NIR-PIT. For example, HER2-targeted NIR-PIT has promising anti-tumour effects in HER2-positive cancers [1]. However, HER2 overexpression ranges from 2.5 to 80% among different cancers [66] and HER2 heterogeneity is well known [67,68]. To overcome this, a replication-deficient adenoviral vector containing a gene that encodes the HER2 extracellular domain (HER2-ECD) was developed, and caused previously HER2 negative transfected cells to express HER2-ECD on the cell surface [69]. Although, the expressed HER2-ECD lacked an intracellular kinase domain and thus, did not trigger HER2 signalling pathways, ADCC activity by trastuzumab was strongly enhanced. HER2-targeted NIR-PIT induced cell death in HER2-ECD-transduced HER2-negative breast cancer cells [70]. Furthermore, HER2-targeted NIR-PIT targeting HER2-ECD inhibited peritoneal metastasis and prolonged survival without severe adverse effects in a mouse model of peritoneal dissemination of HER2-negative gastric cancer [71]. Thus, artificial exogenous receptor domains expressed on the cell surface can become targets for NIR-PIT. In other experiments none of the athymic mice in which HER2-ECD was inserted were cured by HER2-targeted NIR-PIT. However, in the presence of a normal immune system, NIR-PIT can induce massive ICD and patients might acquire immunity against neo-antigens derived from cancer cells killed by NIR-PIT. However, an obvious limitation of this approach is that it is impossible to transfect selectively to only cancer cells. Therefore, although gene transfer technology could expand and potentiate NIR-PIT for target-negative cancers, selective transfection is a barrier to overcome.

### 4.7 Therapeutic evaluation

Monitoring the therapeutic effects in NIR-PIT in real time or immediately after treatment is important in assessing tumour response and determining whether additional treatment is necessary. Preclinically, bioluminescence imaging can evaluate efficacy of NIR-PIT in the acute phase after treatment, but it cannot be translated clinically [72]. ^18^F-fluorodeoxyglucose positron emission tomography (^18^FDG-PET) can be a rapid response marker of treatment success because glucose metabolism in treated tumours was greatly reduced in the early phases after NIR-PIT [73]. Recently, it has been also possible to assess EGFR expression prior to NIR-PIT with immuno-PET imaging using radiolabelled cetuximab [74]. In tumours with high EGFR expression, significantly higher uptake of radiolabelled cetuximab was visually and semi-quantitatively observed compared to tumours with medium or low EGFR expression levels. In the future this method could be used to select patients mostly like to respond to EGFR-targeted NIR-PIT. During NIR-PIT fluorescence imaging of IR700 allows detection of sites of APC binding thus directing light to those fluorescing regions. Since the applied laser light photo-bleaches the IR700, there is a decrease in fluorescence during treatment reaching a minimum plateau after the dye is completely photobleached. The therapeutic effect was correlated with this photo-bleaching such that no further therapeutic effect was expected once the fluorescence reaches the minimum plateau [9]. A clinically approved camera, which is originally designed to detect indocyanine green and typically operates at wavelengths of 830 nm, can detect the fluorescence arising from IR700 during NIR-PIT because the emission spectrum of IR700 extends up to and beyond 830 nm, at least when activated by high intensity laser light. This enables NIR-PIT to be monitored in real time at wavelengths far from the intense laser excitation light at 690 nm which would overwhelm the cameras CCD and this may permit better optimization of NIR light exposure during NIR-PIT [75,76].

## 5 Conclusion

Although most clinical experience has been gained in patients with inoperable HNSCC, NIR-PIT has great potential to become a widely applicable treatment for a variety of cancers. It has the advantages of rapid response with minimal off target effects. When used in combination with immunostimulatory therapies, NIR-PIT may have broader anti-cancer effects throughout the body and may prevent recurrence. Although NIR light does not penetrate deeply in tissue, NIR light can be delivered using both direct surface lighting or via cylindrical diffusing fibres or endoscopes to accommodate various anatomical sites and tumour sizes where a needle or endoscopic device can be inserted. Alternatively, intraoperative or interstitial light delivery can be used for malignancies for which NIR-PIT would otherwise be difficult. Thus, NIR-PIT is a promising candidate for the treatment of HNSCC which serves as an example for other cancers.

## 6 Outstanding questions

NIR-PIT induces a highly selective necrotic/immunogenic cell death for targeted tumour cells, which promotes maturation of DCs and primes cytotoxic T cells to react with cancer-related antigens released from destroyed cancer cells. When NIR-PIT is combined with an immuno-checkpoint inhibitor or NIR-PIT targeting Treg, not only does NIR light expose local tumours but also distant metastatic tumours respond due to the enhanced host tumour immunity induced by the initial anti-tumour NIR-PIT. This “vaccine-like” property of NIR-PIT remains to be explored in human trials.

Another issue is that the optimal treatment regimen of NIR-PIT for different types of tumours is currently under investigation. The degree of targeted molecule expression on the cell surface in treated tumours or distribution of immune cells that have anti-tumorigenic effects and other TILs that have pro-tumorigenic effects in TME could be important factors in selecting ideal patients and ideal combinations of NIR-PIT. However, further investigation is needed to determine an optimal combination strategy with NIR-PIT in each tumour type.

## Search strategy and selection criteria

Data search and selection were identified through searches from PubMed with the following search terms: “near-infrared photoimmunotherapy”, “head and neck cancer”, “monoclonal antibody”, “mouse model”, “epidermal growth factor receptor”, “immune checkpoint inhibitor”, “regulatory T cell”. Only articles published in English between 1989 and 2021 were included.

## Funding

This research was supported by the Intramural Research Program of the National Institutes of Health, ZIA BC011513.

## Contributors

YM and HW wrote the manuscript and designed and produced the figures. PLC and HK outlined and supervised, and edited the draft and approved of the final version to be submitted. All authors approved the final version of the manuscript.

## Declaration of Competing Interest

The authors declare no conflicts of interests.

## References

[1] Mitsunaga M., Ogawa M., Kosaka N., Rosenblum L.T., Choyke P.L., Kobayashi H. (2011). Cancer cell-selective *in vivo* near infrared photoimmunotherapy targeting specific membrane molecules. Nat Med.

[2] Kobayashi H., Choyke PL. (2019). Near-infrared photoimmunotherapy of cancer. Acc Chem Res.

[3] Hanaoka H., Nagaya T., Sato K. (2015). Glypican-3 targeted human heavy chain antibody as a drug carrier for hepatocellular carcinoma therapy. Mol Pharm.

[4] Nagaya T., Nakamura Y., Okuyama S. (2017). Syngeneic mouse models of oral cancer are effectively targeted by anti-CD44-based NIR-PIT. Mol Cancer Res.

[5] Nagaya T., Nakamura Y., Sato K., Harada T., Choyke P.L., Kobayashi H. (2016). Near infrared photoimmunotherapy of B-cell lymphoma. Mol Oncol.

[6] Nagaya T., Nakamura Y., Sato K. (2016). Near infrared photoimmunotherapy with an anti-mesothelin antibody. Oncotarget.

[7] Sato K., Sato N., Xu B. (2016). Spatially selective depletion of tumor-associated regulatory T cells with near-infrared photoimmunotherapy. Sci Transl Med.

[8] Nagaya T., Sato K., Harada T., Nakamura Y., Choyke P.L., Kobayashi H. (2015). Near infrared photoimmunotherapy targeting EGFR positive triple negative breast cancer: optimizing the conjugate-light regimen. PLoS ONE.

[9] Sato K., Ando K., Okuyama S. (2018). Photoinduced ligand release from a silicon phthalocyanine dye conjugated with monoclonal antibodies: a mechanism of cancer cell cytotoxicity after near-infrared photoimmunotherapy. ACS Cent Sci.

[10] Mitsunaga M., Nakajima T., Sano K., Choyke P.L., Kobayashi H. (2012). Near-infrared theranostic photoimmunotherapy (PIT): repeated exposure of light enhances the effect of immunoconjugate. Bioconjug Chem.

[11] Ogawa M., Tomita Y., Nakamura Y. (2017). Immunogenic cancer cell death selectively induced by near infrared photoimmunotherapy initiates host tumor immunity. Oncotarget.

[12] Nagaya T., Friedman J., Maruoka Y. (2019). Host immunity following near-infrared photoimmunotherapy is enhanced with PD-1 checkpoint blockade to eradicate established antigenic tumors. Cancer Immunol Res.

[13] Kobayashi H., Choyke PL. (2016). Super enhanced permeability and retention (SUPR) effects in tumors following near infrared photoimmunotherapy. Nanoscale.

[14] Sano K., Nakajima T., Choyke P.L., Kobayashi H. (2013). Markedly enhanced permeability and retention effects induced by photo-immunotherapy of tumors. ACS Nano.

[15] Sano K., Nakajima T., Choyke P.L., Kobayashi H. (2014). The effect of photoimmunotherapy followed by liposomal daunorubicin in a mixed tumor model: a demonstration of the super-enhanced permeability and retention effect after photoimmunotherapy. Mol Cancer Ther.

[16] Wakiyama H., Furusawa A., Okada R. (2020). Increased immunogenicity of a minimally immunogenic tumor after cancer-targeting near infrared photoimmunotherapy. Cancers.

[17] Sato K., Nagaya T., Mitsunaga M., Choyke P.L., Kobayashi H. (2015). Near infrared photoimmunotherapy for lung metastases. Cancer Lett.

[18] Nagaya T., Okuyama S., Ogata F., Maruoka Y., Choyke P.L., Kobayashi H. (2019). Near infrared photoimmunotherapy using a fiber optic diffuser for treating peritoneal gastric cancer dissemination. Gastric Cancer.

[19] Yamaguchi H., Pantarat N., Suzuki T., Evdokiou A. (2019). Near-infrared photoimmunotherapy using a small protein mimetic for HER2-overexpressing breast cancer. Int J Mol Sci.

[20] Nagaya T., Nakamura Y., Okuyama S. (2017). Near-infrared photoimmunotherapy targeting prostate cancer with prostate-specific membrane antigen (PSMA) antibody. Mol Cancer Res.

[21] Maawy A.A., Hiroshima Y., Zhang Y. (2015). Near infra-red photoimmunotherapy with anti-CEA-IR700 results in extensive tumor lysis and a significant decrease in tumor burden in orthotopic mouse models of pancreatic cancer. PLoS ONE.

[22] Shirasu N., Yamada H., Shibaguchi H., Kuroki M., Kuroki M. (2014). Potent and specific antitumor effect of CEA-targeted photoimmunotherapy. Int J Cancer.

[23] Hiroshima Y., Maawy A., Zhang Y. (2015). Photoimmunotherapy inhibits tumor recurrence after surgical resection on a pancreatic cancer patient-derived orthotopic xenograft (PDOX) nude mouse model. Ann Surg Oncol.

[24] Hanaoka H., Nakajima T., Sato K. (2015). Photoimmunotherapy of hepatocellular carcinoma-targeting Glypican-3 combined with nanosized albumin-bound paclitaxel. Nanomedicine.

[25] Nishimura T., Mitsunaga M., Sawada R. (2019). Photoimmunotherapy targeting biliary-pancreatic cancer with humanized anti-TROP2 antibody. Cancer Med.

[26] Nagaya T., Nakamura Y., Sato K. (2017). Near infrared photoimmunotherapy with avelumab, an anti-programmed death-ligand 1 (PD-L1) antibody. Oncotarget.

[27] Okada R., Maruoka Y., Furusawa A. (2019). The effect of antibody fragments on CD25 targeted regulatory T cell near-infrared photoimmunotherapy. Bioconjug Chem.

[28] Maruoka Y., Furusawa A., Okada R. (2020). Combined CD44- and CD25-targeted near-infrared photoimmunotherapy selectively kills cancer and regulatory T cells in syngeneic mouse cancer models. Cancer Immunol Res.

[29] Maruoka Y., Furusawa A., Okada R. (2020). Near-infrared photoimmunotherapy combined with CTLA4 checkpoint blockade in syngeneic mouse cancer models. Vaccines.

[30] Maruoka Y., Furusawa A., Okada R. (2020). Interleukin-15 after near-infrared photoimmunotherapy (NIR-PIT) enhances T cell response against syngeneic mouse tumors. Cancers.

[31] Jing H., Weidensteiner C., Reichardt W. (2016). Imaging and selective elimination of glioblastoma stem cells with theranostic near-infrared-labeled CD133-specific antibodies. Theranostics.

[32] Okuyama S., Nagaya T., Sato K. (2018). Interstitial near-infrared photoimmunotherapy: effective treatment areas and light doses needed for use with fiber optic diffusers. Oncotarget.

[33] Maruoka Y., Nagaya T., Sato K. (2018). Near infrared photoimmunotherapy with combined exposure of external and interstitial light sources. Mol Pharm.

[34] Maawy A.A., Hiroshima Y., Zhang Y. (2015). Photoimmunotherapy lowers recurrence after pancreatic cancer surgery in orthotopic nude mouse models. J Surg Res.

[35] Sato K., Choyke P.L., Hisataka K. (2021). Selective cell elimination from mixed 3D culture using a near infrared photoimmunotherapy technique. J Vis Exp.

[36] Sato K., Nakajima T., Choyke P.L., Kobayashi H. (2015). Selective cell elimination *in vitro* and *in vivo* from tissues and tumors using antibodies conjugated with a near infrared phthalocyanine. RSC Adv.

[37] Torre L.A., Siegel R.L., Ward E.M., Jemal A. (2016). Global Cancer incidence and mortality rates and trends–an update. Cancer Epidemiol Biomark Prev Publ Am Assoc Cancer Res.

[38] Leemans C.R., Snijders P.J.F., Brakenhoff RH. (2018). Publisher Correction: The molecular landscape of head and neck cancer. Nat Rev Cancer.

[39] Castellsagué X., Quintana M.J., Martínez M.C. (2004). The role of type of tobacco and type of alcoholic beverage in oral carcinogenesis. Int J Cancer.

[40] Chi A.C., Day T.A., Neville BW. (2015). Oral cavity and oropharyngeal squamous cell carcinoma–an update. CA Cancer J Clin.

[41] Epstein J.B., Thariat J., Bensadoun R.J. (2012). Oral complications of cancer and cancer therapy: from cancer treatment to survivorship. CA Cancer J Clin.

[42] Bernier J., Domenge C., Ozsahin M. (2004). Postoperative irradiation with or without concomitant chemotherapy for locally advanced head and neck cancer. N Engl J Med.

[43] Cooper J.S., Pajak T.F., Forastiere A.A. (2004). Postoperative concurrent radiotherapy and chemotherapy for high-risk squamous-cell carcinoma of the head and neck. N Engl J Med.

[44] Martinelli E., De Palma R., Orditura M., De Vita F., Ciardiello F. (2009). Anti-epidermal growth factor receptor monoclonal antibodies in cancer therapy. Clin Exp Immunol.

[45] Grandis J.R., Tweardy DJ. (1993). Elevated levels of transforming growth factor alpha and epidermal growth factor receptor messenger RNA are early markers of carcinogenesis in head and neck cancer. Cancer Res.

[46] Maxwell S.A., Sacks P.G., Gutterman J.U., Gallick GE. (1989). Epidermal growth factor receptor protein-tyrosine kinase activity in human cell lines established from squamous carcinomas of the head and neck. Cancer Res.

[47] Kearsley J.H., Furlong K.L., Cooke R.A., Waters MJ. (1990). An immunohistochemical assessment of cellular proliferation markers in head and neck squamous cell cancers. Br J Cancer.

[48] Ishitoya J., Toriyama M., Oguchi N. (1989). Gene amplification and overexpression of EGF receptor in squamous cell carcinomas of the head and neck. Br J Cancer.

[49] Okada R., Furusawa A., Vermeer D.W. (2021). Near-infrared photoimmunotherapy targeting human-EGFR in a mouse tumor model simulating current and future clinical trials. EBioMedicine.

[50] Ponta H., Sherman L., Herrlich PA. (2003). CD44: from adhesion molecules to signaling regulators. Nat Rev Mol Cell Biol.

[51] Naor D., Nedvetzki S., Golan I., Melnik L., Faitelson Y. CD44 in cancer. Critical reviews in clinical laboratory sciences. 2002;39(6):527-79. 10.1080/10408360290795574 12484499

[52] Chen J., Zhou J., Lu J., Xiong H., Shi X., Gong L. (2014). Significance of CD44 expression in head and neck cancer: a systemic review and meta-analysis. BMC Cancer.

[53] Lee Y., Shin J.H., Longmire M. (2016). CD44+ cells in head and neck squamous cell carcinoma suppress T-cell-mediated immunity by selective constitutive and inducible expression of PD-L1. Clin Cancer Res.

[54] Sakaguchi S., Sakaguchi N., Shimizu J. (2001). Immunologic tolerance maintained by CD25+ CD4+ regulatory T cells: their common role in controlling autoimmunity, tumor immunity, and transplantation tolerance. Immunol Rev.

[55] Kretschmer K., Apostolou I., Jaeckel E., Khazaie K., von Boehmer H. (2006). Making regulatory T cells with defined antigen specificity: role in autoimmunity and cancer. Immunol Rev.

[56] Watanabe Y., Katou F., Ohtani H., Nakayama T., Yoshie O., Hashimoto K. (2010). Tumor-infiltrating lymphocytes, particularly the balance between CD8(+) T cells and CCR4(+) regulatory T cells, affect the survival of patients with oral squamous cell carcinoma. Oral Surg Oral Med Oral Pathol Oral Radiol Endod.

[57] Lu J., Chen X.M., Huang H.R. (2018). Detailed analysis of inflammatory cell infiltration and the prognostic impact on nasopharyngeal carcinoma. Head Neck.

[58] Zou W. (2006). Regulatory T cells, tumor immunity and immunotherapy. Nat Rev Immunol.

[59] Spolski R., Li P., Leonard WJ. (2018). Biology and regulation of IL-2: from molecular mechanisms to human therapy. Nat Rev Immunol.

[60] Sakaguchi S., Sakaguchi N., Asano M., Itoh M., Toda M. (1995). Immunologic self-tolerance maintained by activated T cells expressing IL-2 receptor alpha-chains (CD25). J Immunol.

[61] Boyman O., Kovar M., Rubinstein M.P., Surh C.D., Sprent J. (2006). Selective stimulation of T cell subsets with antibody-cytokine immune complexes. Science.

[62] Okada R., Kato T., Furusawa A. (2021). Local depletion of immune checkpoint ligand CTLA4 expressing cells in tumor beds enhances antitumor host immunity. Adv Ther.

[63] Ito K., Mitsunaga M., Nishimura T., Kobayashi H., Tajiri H. (2016). Combination photoimmunotherapy with monoclonal antibodies recognizing different epitopes of human epidermal growth factor receptor 2: an assessment of phototherapeutic effect based on fluorescence molecular imaging. Oncotarget.

[64] Siddiqui M.R., Railkar R., Sanford T. (2019). Targeting epidermal growth factor receptor (EGFR) and human epidermal growth factor receptor 2 (HER2) expressing bladder cancer using combination photoimmunotherapy (PIT). Sci Rep.

[65] Okada R., Furusawa A., Inagaki F. (2021). Endoscopic Near-infrared Photoimmunotherapy in an orthotopic head and neck cancer model. Cancer Sci.

[66] Oh D.Y., Bang YJ. (2020). HER2-targeted therapies - a role beyond breast cancer. Nat Rev Clin Oncol.

[67] Ishida M., Sekine S., Taniguchi H., Fukagawa T., Katai H., Kushima R. (2014). Consistent absence of HER2 expression, regardless of HER2 amplification status, in neuroendocrine carcinomas of the stomach. Histopathology.

[68] Lee H.J., Seo A.N., Kim E.J. (2014). HER2 heterogeneity affects trastuzumab responses and survival in patients with HER2-positive metastatic breast cancer. Am J Clin Pathol.

[69] Yoshida R., Tazawa H., Hashimoto Y. (2012). Mechanism of resistance to trastuzumab and molecular sensitization via ADCC activation by exogenous expression of HER2-extracellular domain in human cancer cells. Cancer Immunol Immunother CII.

[70] Shimoyama K., Kagawa S., Ishida M. (2015). Viral transduction of the HER2-extracellular domain expands trastuzumab-based photoimmunotherapy for HER2-negative breast cancer cells. Breast Cancer Res Treat.

[71] Ishida M., Kagawa S., Shimoyama K. (2016). Trastuzumab-based photoimmunotherapy integrated with viral HER2 transduction inhibits peritoneally disseminated HER2-negative cancer. Mol Cancer Ther.

[72] Maruoka Y., Nagaya T., Nakamura Y. (2017). Evaluation of early therapeutic effects after near-infrared photoimmunotherapy (NIR-PIT) using luciferase-luciferin photon-counting and fluorescence imaging. Mol Pharm.

[73] Sano K., Mitsunaga M., Nakajima T., Choyke P.L., Kobayashi H. (2013). Acute cytotoxic effects of photoimmunotherapy assessed by 18F-FDG PET. J Nucl Med.

[74] Yamaguchi A., Achmad A., Hanaoka H. (2019). Immuno-PET imaging for non-invasive assessment of cetuximab accumulation in non-small cell lung cancer. BMC Cancer.

[75] Inagaki F.F., Fujimura D., Furusawa A. (2021). Fluorescence imaging of tumor-accumulating antibody-IR700 conjugates prior to near-infrared photoimmunotherapy (NIR-PIT) using a commercially available camera designed for indocyanine green. Mol Pharm.

[76] Okuyama S., Fujimura D., Inagaki F. (2021). Real-time IR700 fluorescence imaging during near-infrared photoimmunotherapy using a clinically-approved camera for indocyanine green. Cancer Diagn Progn.

